# Nobiletin in Cancer Therapy: How This Plant Derived-Natural Compound Targets Various Oncogene and Onco-Suppressor Pathways

**DOI:** 10.3390/biomedicines8050110

**Published:** 2020-05-05

**Authors:** Milad Ashrafizadeh, Ali Zarrabi, Sedigheh Saberifar, Farid Hashemi, Kiavash Hushmandi, Fardin Hashemi, Ebrahim Rahmani Moghadam, Reza Mohammadinejad, Masoud Najafi, Manoj Garg

**Affiliations:** 1 Department of Basic Science, Faculty of Veterinary Medicine, University of Tabriz, Tabriz 5166616471, Iran; dvm.milad73@yahoo.com; 2 Sabanci University Nanotechnology Research and Application Center (SUNUM), Tuzla, Istanbul 34956, Turkey; alizarrabi@sabanciuniv.edu; 3 Department of Basic Sciences, Faculty of Veterinary Medicine, Shahid Chamran University of Ahvaz, Ahvaz 6135783151, Iran; sedigheh.saberifar@gmail.com; 4 DVM. Graduated, Young Researcher and Elite Club, Kazerun Branch, Islamic Azad University, Kazeroon 7319846451, Iran; faridhashemi172@gmail.com; 5 Department of Food Hygiene and Quality Control, Division of Epidemiology, Faculty of Veterinary Medicine, University of Tehran, Tehran 1417414418, Iran; houshmandi.kia7@ut.ac.ir; 6 Student Research Committee, Department of Physiotherapy, Faculty of Rehabilitation, Ahvaz Jundishapur University of Medical Sciences, Ahvaz 6135715749, Iran; Fardinhashemi6@gmail.com; 7 Student Research Committee, Department of Anatomical Sciences, School of Medicine, Shiraz University of Medical Sciences, Shiraz 7134814336, Iran; Ebrahimrahmani1374@gmail.com; 8 Neuroscience Research Center, Institute of Neuropharmacology, Kerman University of Medical Sciences, Kerman 7619813159, Iran; 9 Radiology and Nuclear Medicine Department, School of Paramedical Sciences, Kermanshah University of Medical Sciences, Kermanshah 6715847141, Iran; 10 Amity Institute of Molecular Medicine and Stem Cell Research (AIMMSCR), Amity University Uttar Pradesh, Sector-125, Noida-201313, India

**Keywords:** nobiletin, flavonoid, citrus, cancer therapy, signaling pathway, herbal compound

## Abstract

Cancer therapy is a growing field, and annually, a high number of research is performed to develop novel antitumor drugs. Attempts to find new antitumor drugs continue, since cancer cells are able to acquire resistance to conventional drugs. Natural chemicals can be considered as promising candidates in the field of cancer therapy due to their multiple-targeting capability. The nobiletin (NOB) is a ubiquitous flavone isolated from Citrus fruits. The NOB has a variety of pharmacological activities, such as antidiabetes, antioxidant, anti-inflammatory, hepatoprotective, and neuroprotective. Among them, the antitumor activity of NOB has been under attention over recent years. In this review, we comprehensively describe the efficacy of NOB in cancer therapy. NOB induces apoptosis and cell cycle arrest in cancer cells. It can suppress migration and invasion of cancer cells via the inhibition of epithelial-to-mesenchymal transition (EMT) and EMT-related factors such as TGF-β, ZEB, Slug, and Snail. Besides, NOB inhibits oncogene factors such as STAT3, NF-κB, Akt, PI3K, Wnt, and so on. Noteworthy, onco-suppressor factors such as microRNA-7 and -200b undergo upregulation by NOB in cancer therapy. These onco-suppressor and oncogene pathways and mechanisms are discussed in this review.

## 1. Introduction

Recently, nutritionists have been interested in recommending plants and fruits in the treatment of different illnesses [1,2]. This suggestion is due to the presence of beneficial natural chemicals in plants and fruits and, also, their metabolites, which exert health-promoting effects after being absorbed in the body [3,4,5,6,7,8,9,10]. It has been demonstrated that the identification, isolation, and purification of these natural chemicals may be a milestone in the treatment of diseases, particularly in human malignancies [11,12,13,14,15,16,17,18,19,20]. A high number of studies have focused on cancer therapy using plant-derived natural compounds [6,13,14,15,16,17,18,19,20]. In this review, we demonstrate the potential of nobiletin (NOB) in cancer therapy based on the newly published articles.

## 2. Sources of NOB

The NOB, as a polymethoxyflavone (PMF), was named after *Citrus nobilis* [21]. NOB is a ubiquitous flavone extensively derived from the peel of *Citrus* fruits [22]. Interestingly, NOB can be isolated from a variety of *Citrus* fruits, including mandarin oranges (*Citrus reticulate*), sweet oranges or Valencia oranges (*Citrus sinesis*), Miaray mandarins (*Citrus miaray*), flat lemons or Hayata (*Citrus depressa*), tangerines (*Citrus tangerine*), bitter oranges (*Citrus aurantium*), Unshu Mikans or Satsuma mandarins (*Citrus Unshiu arnicia indica*), Cleopatra mandarins (*Citrus reshni*), mandarin oranges (*Citrus tachibana*), Koji oranges (*Citrus leiocarpa*), Natsu Mikans (*Citrus tardira*), Jimikan (*Citrus succosa*), kinokuni mandarins (*Citrus kinokuni*), Fukushu (*Citrus erythrose*), Supkat (*Citrus sunki*), and hybrids of mandarin orange with pomelo (*Citrus deliciosa*) [22,23,24,25,26,27,28]. This shows that NOB is abundantly found in nature, and using it in the treatment of diseases is a cost-effective approach. Among the aforementioned plants, *Citrus tangerine* has the highest concentration of NOB, leading to its application in disease therapy [29]. Several methods are applied to isolate PMF from orange peel, such as supercritical fluid extraction, microwave-assisted extraction, and the Soxhlet method, enabling us to obtain high contents of this extract [30]. At the final step of extraction, carbon dioxide and ethanol are used to concentrate bioactive compounds [31]. The highest yield of NOB is observed at a temperature of 80 °C, the pressure of 30 MPa, and an optimum sample particle size of 375 μm [32]. In addition to these conventional methods, NOB can be isolated by total synthesis of over eleven steps [33]. The NOB has a molecular weight of 402.39, and its chemical and molecular formula are 5,6,7,8,3^/^,4^/^-hexamethoxy flavone, and C_21_H_22_O_8_, respectively [34]. Chromene and arene rings of NOB are at the same plane. The C atoms of two methoxy groups in the arene ring are at the same plane. However, C atoms of four methoxy groups linking to a chromene ring may not necessarily be in parallel [35].

## 3. Bioavailability of NOB

Although studies exhibit that NOB is exclusively found in nature and various *Citrus* plants, some restrictions have reduced NOB potential. It has been demonstrated that NOB has poor solubility in water (1–5 μg/mL) and minimal oral bioavailability (˂1%), resulting in a decrease in its therapeutic and biological activities [36]. It is worth mentioning that, after ingestion, NOB undergoes many alterations to produce metabolites [37,38]. The kind of metabolite depends on the species of *Citrus* plant [22]. Three common metabolites of NOB include 3^/^-demethylnobiletin (3^/^-DMN), 4^/^-DMN, and 3^/^,4^/^-DMN [39,40]. A study has investigated the amount of aforementioned metabolites in mice after 20 weeks of daily feeding of 500 ppm NOB as 3.28 (3^/^-DMN), 24.13 (4^/^-DMN), and 12.03 (3^/^,4^/^-DMN) nmol/g. Interestingly, the bioavailability of NOB was reported as 2.03 nmol/g, which was lower compared to its metabolites [41]. This shows that NOB is immediately metabolized in the body into its metabolites. The metabolism of NOB comprises two phases, including phase I and phase II metabolism. The cytochrome P450 participates in phase I demethylation of NOB [42]. The CYP1A1, CYP1A2, CYP1B, and CYP3A5 are involved in the conversion of NOB into 3/DMN, while only CYP1A1 and CYP1A2 contribute to the transformation of 3^/^-DMN into 3^/^,4^/^-DMN [43]. The phase II metabolism of NOB occurs in the small intestine by sulfation or glucuronidation [44]. As a consequence of the rapid metabolism of NOB and its poor bioavailability, studies have focused on improving NOB bioavailability using various methods. Recently, an ionic liquid containing choline and geranic acid (CAGE) has been developed for promoting NOB bioavailability. The in vitro and in vivo experiments have demonstrated the capability of CAGE in enhancing NOB bioavailability. The enhanced bioavailability of NOB by CAGE is due to the multipoint hydrogen bonding between NOB and CAGE. The CAGE not only elevates the transdermal absorption of NOB but also increases the bioavailability of NOB after oral administration by 20 times [45]. The plant exine capsules can also be considered as a potential strategy in improving NOB bioavailability, since plant exine capsules have high loading capacity (770 ± 40 mg/g) and provide the prolonged release of NOB [46]. It is worth mentioning that nanostrategies are also promising candidates in enhancing NOB bioavailability. It is said that NOB-loaded nanoemulsions are able to enhance the therapeutic capacity of NOB [47]. Micelles are other nanoparticles that have been used in the delivery of NOB for bone loss treatment with excellent features such as low particle size (124 nm), high loading capacity (7.6%), and great entrapment efficiency (76.34%) [48]. However, we are at the beginning point of NOB delivery, and more studies are required to develop novel carriers for the delivery of NOB.

## 4. Therapeutic and Biological Activities of NOB

The interest directed towards NOB emanates from its efficacy in the treatment of different diseases. Studies have demonstrated that NOB has a variety of therapeutic and biological activities, including antidiabetic [49], antioxidant [50], osteoprotective [51], anti-inflammatory [52], hepatoprotective [53], cardioprotective [54,55], and neuroprotective [56], as well as improving metabolic disorders [57]. Notably, recent studies have shown the role of molecular signaling pathways involved in the protective effects of NOB in various diseases. Diabetes mellitus (DM) is a chronic metabolic disorder that glucose uptake undergoes impairment [58]. It is held that inflammatory factors participate in glucose uptake interference [59]. The administration of NOB (50 mg/kg) remarkably improves glucose resistance by inhibition of the NF-κB signaling pathway, as a factor involved in inflammation to suppress tumor necrosis factor (TNF)-mediated glucose uptake disruption [60]. Although iron is a vital element for physiological processes such as hemoglobin synthesis and DNA replication [61], increasing evidence shows that iron overload is associated with the elevated generation of reactive oxygen species (ROS) that, in turn, induce cell and tissue damages [62,63,64,65,66,67,68]. The NOB is suggested to be a potential agent in fighting against iron overload. The administration of NOB inhibits mitochondrial-mediated apoptosis via reducing ROS generation to attenuate vascular endothelium injury caused by iron overload [69]. Interleukin-21 (IL-21), produced by stimulated CD4+ T immune cells [70], plays a significant role in the progression of rheumatoid arthritis (RA) via induction of inflammatory factors and matrix metalloproteinases (MMPs) such as MMP-3 and MMP-13 [71]. A newly published article (2020) has examined the efficacy of NOB in RA therapy. It seems that IL-21 binds to its receptor to elevate ROS generation. On one hand, ROS induces mitochondrial dysfunction, and on the other hand, ROS stimulates the JAK1/STAT3 axis to stimulate MMP-3 and -13 and inflammatory factors including TNF-α and IL-6. The NOB disrupts the IL-6 attachment into its receptor to interfere with the aforementioned axis, leading to the alleviation of RA [72]. The NOB not only is beneficial in the management and treatment of DM, but also, it can be recommended for the prevention of DM, since it is capable of decreasing insulin resistance, obesity, dyslipidemia, and hepatic steatosis [73]. Nowadays, a high number of people are searching for promoting their longevity. The NOB is suggested to be beneficial in this case. An experiment on *Caenorhabditis elegans* exhibits that NOB has great antitumor activity and can enhance the lifespan by attenuation of heat shock and ultraviolet radiation [74]. We earlier mentioned that NOB is advantageous in suppressing iron overload-mediated oxidative stress. It is said that the high antioxidant activity of NOB results from improving the antioxidant defense system by targeting nuclear factor erythroid 2-related factor 2 (Nrf2) [75]. The activation of Nrf2 reduces ROS generation and oxidative stress via stimulation of superoxide dismutase, heme oxygenase-1, and NADPH quinone oxidoreductase 1 [75]. By induction of Nrf2, NOB decreases ROS levels to ameliorate hepatorenal toxicity [75]. Another pathological condition that can be alleviated by NOB is ischemic/reperfusion (I/R) injury, a condition involved in enhancing oxidative stress and inflammation [76]. Notably, NOB dually enhances the activity of antioxidant enzymes such as catalase and glutathione peroxidase (GSH-PX), whereas it diminishes the concentration of IL-6 and TNF-α, leading to amelioration of I/R-mediated inflammation and oxidative stress [77]. It has been demonstrated that antioxidant and anti-inflammatory activities of NOB during I/R is mediated by mitogen-activated protein kinase (MAPK) induction [78]. It is said that NOB is capable of the treatment of metabolic disorders and recovering cholesterol balance via the stimulation of bile acid synthesis [79]. In Table 1, we have summarized the therapeutic and biological activities of NOB. These studies highlight the potential of NOB in disease treatment and its protective effects. The newly published articles have shed some light on the capability of NOB in cancer therapy [80,81]. In the present review, we attempt to mechanistically examine the efficiency of NOB in cancer therapy by focusing on molecular pathways and mechanisms.

## 5. Potential Role of NOB in Human Malignancies

### 5.1. Nobiletin and Chemotherapy

The estimates demonstrate that finding effective treatments for cancer is of importance due to the enhanced incidence rate of this life-threatening disorder. Chemotherapy is one of the most common ways in cancer therapy, and due to its minimally invasive nature, scientists have focused on cancer therapy using chemotherapeutic agents. However, a high number of patients with cancer are directed towards death due to chemotherapy failure caused by multidrug resistance (MDR) [91]. The transport-based classical and nonclassical MDR phenotypes are responsible for the cellular mechanisms of drug resistance [92]. The P-glycoprotein (P-gp) is a member of the ATP-binding cassette (ABC)-family efflux transporters encoded by the MDR1 gene. A variety of studies have evaluated the role of P-gp in different cancers. It is said that enhanced expression of P-gp elevates malignant behavior and the progression of cancer cells via the stimulation of epithelial-to-mesenchymal transition (EMT) [93]. The antitumor drugs exert their inhibitory effect on the proliferation and viability of MDR cancer cells via the inhibition of P-gp [94]. Consequently, scientists in the field of medicinal chemistry have attempted to develop novel drugs targeting and suppressing P-gp activity in cancer cells [95]. It has been demonstrated that NOB targets P-gp in cancer therapy. A newly published experiment developed a derivative of NOB to enhance its solubility and antitumor activity. This agent, known as compound 29d, can increase the accumulation of paclitaxel (PTX) in tumor cells (lung cancer, A549 cells) via reducing the P-gp activity [96]. On the other hand, increasing evidence demonstrates that the Nrf2/PI3K/Akt and the extracellular signal-regulated kinase (ERK) pathway pathways can stimulate chemoresistance [97]. The compound 29d administration is associated with the downregulation of ERK. Besides, NOB can inhibit the PI3K/Akt signaling pathway via Nrf2 downregulation [96]. This study highlights the fact that NOB and its derivatives target different pathways to sensitize cancer cells into chemotherapy. Similarly, another study evaluates the efficacy of NOB in enhancing the antitumor activity of PTX. The same molecular pathways were investigated. It is held that, by downregulation of Nrf2 and Akt and ERK phosphorylation, NOB sensitizes MDR lung cancer cells into PTX-mediated apoptosis [96]. Although these two studies showed similar findings of the Akt and Nrf2 pathways, the latter study exhibits that, in increasing PTX efficacy for the elimination of lung cancer cells, NOB does not affect P-gp activity. This difference is because compound 29d is a derivative of NOB with higher antitumor activity. However, more studies are required to examine this controversy. An experiment reveals that NOB can significantly decrease the viability and survival of cancer cells in a dose-dependent manner, but it does not affect the cell cycle. It is said that a combination of NOB and cisplatin has a more inhibitory effect on the viability of thyroid cancer cells compared to NOB or cisplatin alone. It is worth mentioning that, in reducing the viability of cancer cells, NOB does not negatively affect normal cells [98], making it a suitable option in chemotherapy. Sorafenib is a receptor tyrosine kinase (RTK) inhibitor approved by the Food and Drug Administration (FDA). The sorafenib is extensively applied in the treatment of different cancers with high efficacy [99,100]. The NOB can be co-administered with sorafenib to elevate its antitumor activity. A combination of NOB and sorafenib remarkably diminishes the proliferation and viability of prostate cancer cells by the stimulation of apoptosis and cell cycle arrest via enhancing the expression of Bax, Rb1, and CDKN1A [101]. The multidrug resistance-associated protein 1 (MRP1), known as ABCC1, was first recognized in lung cancer cells that had no expression of ABCB1 (MDR1 or P-gp) [102]. It has been reported that MRP1 stimulates chemoresistance via neuroblastoma-derived MYC (MYCN) [103,104,105]. The fibrous sheath-interacting protein 1 (FSIP1) is able to stimulate chemoresistance via MRP1 induction [106]. Notably, miR-7 functions as an onco-suppressor miR to sensitize breast cancer cells into chemotherapy via MRP1 inhibition [107]. The NOB follows the same route in sensitizing lung cancer cells into adriamycin chemotherapy. NOB enhances adriamycin accumulation in cancer cells via downregulation of MRP1, leading to the induction of apoptosis [108]. On the other hand, the Wnt/β-catenin signaling pathway plays a significant role in cancer development [109,110,111]. Antitumor drugs such as echinacoside diminish the malignancy and proliferation of cancer cells via Wnt inhibition [112]. Besides, miR-455-3p inhibits EMT and the invasion of cancer cells via downregulation of Wnt/β-catenin [112]. The Akt can reduce the activity of GSK-3β via its phosphorylation at serine9 to ensure the nuclear translocation of β-catenin and activation of the Wnt signaling pathway, whereas active GSK-3β inhibits the nuclear translocation of β-catenin via ubiquitination [113]. In enhancing the antitumor activity of adriamycin, NOB inhibits Akt to suppress the Wnt/β-catenin signaling pathway via elevating GSK-3β activity, leading to the reduced viability and proliferation of lung cancer cells [108]. One of the most well-known and studied signaling pathways is the PI3K/Akt/mTOR signaling pathway [114,115,116,117,118]. This axis participates in cell proliferation and metabolism. Consequently, tumor cells prefer to activate the PI3K/Akt/mTOR signaling pathway in enhancing their survival and growth [119]. The antitumor drugs negatively affect the PI3K/Akt/mTOR signaling pathway to suppress proliferation. For instance, sanggenol triggers apoptosis and cell cycle arrest in cancer cells via inhibition of the PI3K/Akt/mTOR axis [120]. Pitavastatin limits the migration and proliferation of cancer cells by the inhibition of angiogenesis via PI3K/Akt/mTOR downregulation [121]. In sensitizing cancer cells with oxaliplatin and reducing the viability and proliferation of colorectal cancer (CRC) cells, NOB inhibits the PI3K/Akt/mTOR signaling pathway, resulting in the induction of apoptosis via reducing the expression of Bcl-2 and enhancing the expression of Bax and caspase-3. The PI3K induces the mTOR signaling pathway via Akt phosphorylation. This axis results in cell proliferation and the growth of cancer cells. Targeting this pathway by NOB mediates its antitumor activity [122]. Overall, the studies exhibit that NOB not only can enhance chemosensitivity via the inhibition of P-gp, but also, it can suppress oncogene signaling pathways such as Nrf2 and Akt/ERK to inhibit cancer progression and sensitize them into chemotherapy [123]. It is worth mentioning that colonic metabolites of NOB such as 3/-DMN, 4/-DMN, and 3/,4/-DMN have chemo-preventive effects. In respect to the higher bioavailability of 3/-DMN, 4/-DMN, and 3/,4/-DMN compared to NOB, they can considerably suppress the invasion, proliferation, and survival of colon cancer cells via the stimulation of apoptosis and cell cycle arrest [41], making them suitable options in chemotherapy.

### 5.2. Relation between NOB and Metastasis

Metastasis is an increasing challenge in enhancing the overall survival rate of patients with cancer and associated with poor prognosis [124]. Earlier, we had described that EMT is a factor that enhances the metastasis and migration of cancer cells. MMPs are also able to provide the metastasis and invasion of tumor cells via the degradation of the extracellular matrix (ECM) [125,126]. Among MMPs, MMP-2 and MMP-9 are important due to their ability in the degradation of major components of the ECM, including gelatin, collagen, and laminin [127]. The overexpressions of MMP-2 and MMP-9 are related to the undesirable prognosis of patients with cancer [128]. Noteworthy, several molecular signaling pathways such as NF-κB, specificity protein-1 (SP-1), cAMP response element-binding protein (CREB), ERK, and JNK can regulate MMP expression [129,130,131]. The NOB suppresses the motility and invasion of cancer cells via the downregulation of MMP-2 and MMP-9. The investigation of molecular signaling pathways exhibits that NOB reduces MMP-2 and MMP-9 expressions through the inhibition of ERK and JNK pathways and downstream targets such as NF-κB, CREB, and SP-1. Overall, CREB and SP-1 interactions are necessary for MMP-2 expression, while NF-κB and SP-1 interactions are responsible for MMP-9 expression. In this way, JNK and ERK act as upstream mediators in the stimulation of CREB/SP-1/MMP-2 and NF-κB/SP-1/MMP-9 signaling pathways. In the inhibition of osteosarcoma migration, NOB negatively affects the aforementioned signaling pathways. NOB indirectly affects the target involved in the metastasis of osteosarcoma (MMP-2 and MMP-9) and, by downregulation of their upstream modulators, paves the way for the inhibition of metastasis and improving prognosis [132].

### 5.3. Head and Neck Cancers

The poly (ADP-ribose) polymerases (PARPs) are enzymes involved in catalyzing the poly (ADP-ribosylation (PARylation) [133]. Among the 18 members of PARPs, PARP-1/2 contribute to the repair of DNA injury [134]. The sirtuin 1 (SIRT1) is suggested to be a downstream target of PARP [135]. The implication of PARP/SIRT1 in cancer has been explored [134]. Inhibition of PARP2 by onco-suppressor miR-383 diminishes the progression of cancer cells and sensitizes them into cell death [136]. Interestingly, the administration of NOB is correlated with the downregulation of PARP2. As a downstream target of PARP2, SIRT1 undergoes upregulation that, in turn, induces the AMPK signaling pathway to stimulate apoptosis in nasopharyngeal carcinoma cells and to suppress their proliferation [137]. It is well-understood that EMT enhances the migration and metastasis of cancer cells. During this process, epithelial cells are transformed into mesenchymal ones that have high migratory and metastatic capabilities. In this way, E-cadherin as an epithelial protein undergoes downregulation, while an increase occurs in mesenchymal markers such as N-cadherin and vimentin [138,139,140,141]. Consequently, targeting this mechanism remarkably reduces the invasion of cancer cells. The administration of NOB is related to the downregulation of TGF-β and Slug, as upstream mediators involved in EMT induction, resulting in increasing E-cadherin and occluding levels and decreasing N-cadherin and fibronectin levels. The examination of molecular pathways demonstrates that TGF-β induces the nuclear translocation of β-catenin in EMT induction, and by the inhibition of TGF-β, NOB suppresses the EMT of glioma cells [142].

### 5.4. Thoracic Cancers

As we mentioned in the introduction section, NOB undergoes a transformation in the body and produces three common metabolites, including 3^/^-DMN, 4^/^-DMN, and 3^/^,4^/^-DMN. Interestingly, a newly published study has investigated the efficacy of NOB and its common metabolites in the treatment of lung cancer. This study displays that NOB and its metabolites have a great potential to suppress lung cancer tumorigenesis, but 4^/^-DMN and 3^/^,4^/^-DMN possess higher antitumor activity compared to NOB. The antitumor activity of NOB and its metabolites is mediated by their effect on the stimulation of apoptosis and cell cycle arrest via the overexpression of p21, CDK1, cyclin D1, CDK6, CDK4, Bax, and caspase, as well as PARP [143]. The accumulating data demonstrates that MMPs play a pivotal role in the migration and metastasis of cancer cells via degradation of the base membrane [144]. To suppress the migration and invasion of breast cancer cells, NOB downregulates the expression of MMP-2 and MMP-9 [145]. In respect to the role of MAPK in cell proliferation and apoptosis [146], targeting this pathway is of importance in cancer therapy. It seems that the stimulation of MAPK can inhibit both the migration and growth of cancer cells [147]. In breast cancer cells, NOB enhances p38 MAPK expression and its phosphorylation to inhibit breast cancer progression [145]. In the introduction section, we mentioned that the antioxidant activity of NOB relies on Nrf2 activation. However, the story is completely different in cancer cells. It is held that Nrf2 activation can ensure the proliferation of tumor cells and induces chemoresistance [148]. In breast cancer cells, NOB supplementation reduces the expression of Nrf2 and inhibits the nuclear translocation of Nrf2 to suppress breast cancer proliferation [145]. This study highlights the fact that NOB is able to simultaneously target different molecular pathways that make it an appropriate option in cancer therapy. It seems that CD36 participates in tumor metastasis via the regulation of lipid metabolism. The interaction between CD36 and TGF-β stimulates EMT mechanisms to enhance the migration and metastasis of cancer cells [149]. CD36 overexpression is associated with the poor prognosis and resistance of cancer cells into chemotherapy-mediated apoptosis [147]. The effect of CD36 on the migration and invasion of cancer cells is due to the downregulation of E-cadherin and β-catenin [150]. On the other hand, stimulation of the STAT3 and NF-κB signaling pathways mediates the angiogenesis of cancer cells, and the inhibition of STAT3 can suppress migration [151]. Normally, CD36 stimulates the nuclear translocation of STAT3 and NF-κB to induce angiogenesis. The activation of NF-κB occurs as a result of the nuclear translocation of STAT3. The administration of NOB restricts the angiogenesis, migration, and proliferation of breast cancer cells via inhibition of the CD36/STAT3/NF-κB axis [152]. Interestingly, studies have shown that CYP1 enzymes contribute to the bioactivation of flavonoids and mediating their antitumor activity [153]. This story is also true for NOB. The cytochrome P450 CYP1 plays a significant role in the bioactivation of NOB in breast cancer cells [154]. By bioactivation of NOB via CYP1, an increase occurs in its capability in the induction of apoptosis and cell cycle arrest at the G1 phase. Using a CYP1 inhibitor remarkably reduces antitumor activity against breast cancer cells [155], exhibiting that NOB metabolism by cytochrome P450 CYP1 can be targeted in further studies. Aromatase is another enzyme involved in estrogen biosynthesis [156]. The aromatase is a key member of cytochrome P450 CYP1 capable of converting androstenedione into estrone (E1) [157]. The expressions and activities of aromatase demonstrate an increase in patients with breast cancer [158,159]. So, aromatase is an oncogene factor in breast cancer, and its activity should be inhibited. Notably, the administration of NOB at high doses (10 μM) enhances the expression and activity of aromatase, while low doses (1 μM) inhibits the aromatase activity. This study highlights the fact that, in targeting the metabolism of breast cancer cells, low doses of NOB should be used [160]. In respect to the role of EMT in the migration and malignant behavior of cancer cells, much attention has been directed towards identifying molecular signaling pathways related to EMT induction [161,162,163,164,165]. The TGF-β1 is able to stimulate EMT by the phosphorylation of Smad2 and Smad3 and the subsequent formation of a complex with Smad4. Then, the Smad2/3/4 complex translocates into the nucleus to induce an EMT [166]. So, targeting Smads is of importance in suppressing metastasis. In lung cancer cells that have high metastatic ability and demonstrate migration into neighboring cells and tissues, reducing metastatic factors can alleviate poor prognosis. In this way, NOB disrupts TGF-β1 and Smad3 in EMT induction. As a consequence, the ability of cancer cells in migration undergoes downregulation [167]. The Notch1 is an oncogene factor that its role in different cancers has been evaluated. It is said that tumor-educated B (TEB) cells are able to enhance cancer progression via the activation of IL-1β/HIF-2α and subsequent induction of Notch1 [168]. As a histone methyltransferase, G9a increases the malignant behavior of cancer cells via Notch1 overexpression [169]. These studies show that Notch1 should be inhibited in cancer therapy. The NOB supplementation suppresses hypoxia-mediated EMT in lung tumor cells via the downregulation of Notch1. In this way, NOB inhibits downstream targets of Notch1 such as Hey1 and Hes1 and, also, Jagged1/2. It is worth mentioning that, by the downregulation of Notch1, EMT-related factors including Twist1, Snail1, and ZEB1/2 undergo a decrease [170]. On the other hand, miR-200b is an onco-suppressor factor that inhibits downstream targets such as laminin subunit alpha 4 (LAMA4) to reduce the invasion and proliferation of cancer cells [171]. During the metastasis of cancer cells, the expression of miR-200b undergoes downregulation [172]. So, antitumor drugs should elevate miR-200b expression. NOB enhances the expression of miR-200b in hypoxic conditions to suppress the EMT-mediated metastasis of lung cancer cells [170].

### 5.5. Gynecological Cancers

The programmed cell death (PCD) includes the apoptosis, pyroptosis, and autophagy that are made by caspases, lysosomal proteases, and endonucleases [173,174,175,176]. During recent decades, much attention has been directed towards targeting three major arms of PCD in cancer therapy. The pyroptosis participates in cell death via the induction of DNA fragmentation. GSDMD and GSDME are members of pyroptosis [177]. The NOB enhances ROS generation to stimulate mitochondrial dysfunction via decreasing the mitochondrial membrane potential, leading to autophagy activation. It seems that this pathway upregulates GSDMD/GSDME to trigger pyroptosis, resulting in a diminution in the viability of ovarian cancer cells [178]. The molecular biologists who work in the field of cancer believe that cancer cells can obtain resistance to chemotherapy and enhance their proliferation using autophagy induction [179,180]. Accumulating data has investigated the role of autophagy and its regulation in chemoresistance [181,182]. The TSPAN9 is a transmembrane protein that can stimulate the chemoresistance of cancer cells through autophagy induction [183]. It seems that there is a dual relationship between EMT and autophagy. By the inhibition of autophagy, the malignant behavior of cancer cells undergoes downregulation to sensitize cancer cells into chemotherapy [184]. Overall, studies are in agreement with the fact that autophagy activation may mediate chemoresistance [185]. In ovarian cancer cells, NOB targets autophagy to stimulate cell cycle arrest and apoptosis. Via upregulation of the Akt signaling pathway, NOB inhibits autophagy to sensitize cancer cells into apoptosis. Autophagy functions as a pro-survival mechanism, and its inhibition by NOB triggers the intrinsic pathway of apoptosis via the induction of caspase-9, caspase-3, and PARP [186].

### 5.6. Urological Cancers

**Figure 1:**
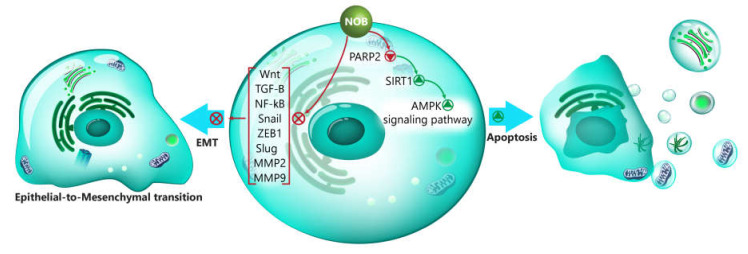
The involvement of signaling pathways in the regulation of EMT by NOB. AMPK, AMP-activated protein kinase; SIRT1, sirtuin 1; PARP2, poly (ADP-ribose) polymerase 2; NOB, nobiletin; Wnt, Wingless-related integration site; TGF-β, transforming growth factor-β; NF-κB, nuclear factor-kappa B; ZEB1, zinc finger E-box binding homeobox 1; MMP-2, matrix metalloproteinase-2; MMP-9, matrix metalloproteinase-9; and EMT, epithelial-to-mesenchymal transition.

The Toll-like receptors (TLRs) are expressed in a variety of immune cells, including macrophages, dendritic cells, and natural killer (NK) cells. There are 10 distinct types of TLRs (TLR1-10), and they undergo induction by endogenous or exogenous ligands carrying pathogen-associated molecular patterns (PAMPs) regions [187]. Although TLRs are involved in the immune response, TLR2, TLR4, and TLR9 contribute to cancer proliferation and progression [188]. So, scientists should consider the oncogene role of TLR4 and TLR9 in cancer cells. It seems that TLR4/MyD88/NF-κB and TLR4/TRIF/IRF3 are involved in the production of inflammatory cytokines after the identification of lipopolysaccharide (LPS) [189]. In prostate cancer cells, NOB exerts an inhibitory impact on their growth and proliferation. The investigation of molecular pathways shows that NOB is able to inhibit TLR9/IRF7 and TLR4/TRIF/IRF3 in suppressing the proliferation and growth of cancer cells [190]. In respect to the role of inflammation in cancer growth and the involvement of TLRs in the production of inflammatory factors such as interferon-γ (IFN-γ) and IFN-β, NOB inhibits prostate cancer growth by its anti-inflammatory activity. In the intrinsic pathway of apoptosis, the mitochondrion and endoplasmic reticulum (ER) play significant roles [191,192]. External stimuli such as ROS are able to disrupt the mitochondrial membrane integrity via the upregulation of Bax and downregulation of Bcl-2. Following cytochrome c release into the cytosol, the caspase-9 and caspase-3 are activated to induce apoptotic cell death [193,194]. The main function of ER is to modulate protein synthesis, protein folding, and calcium homeostasis [195]. The accumulation of unfolded proteins stimulates ER stress, leading to the activation of apoptosis, unfolded protein response (UPR), and ER-associated degradation (ERAD). The PKR-like ER-associated kinase (PERK), inositol requiring enzyme-1α (IRE1α), and activating transcription 6 (ATF6) are three major arms of the UPR that can either stimulate autophagy or apoptosis [196,197,198]. On the other hand, the PI3K/Akt/mTOR signaling pathway regulates apoptosis and cell proliferation [199]. The administration of NOB targets all of these pathways and mechanisms. By downregulation of the PI3K/Akt/mTOR axis, NOB inhibits the proliferation and growth of bladder cancer cells. NOB induces mitochondrial dysfunction to release cytochrome C, resulting in stimulation of proapoptotic factors caspase-3, caspase-9, Bad, and Bax. Besides, NOB triggers the PERK/elF2α/ATF4/CHOP axis through ER stress to activate apoptosis. These molecular pathways and mechanisms targeted by NOB reduce the invasion and proliferation of bladder cancer cells [200]. The signal transducer and activator of transcription 3 (STAT3) and YY1-associated protein 1 (YY1AP1) are two oncogene factors in cancer cells [201,202,203,204]. The YYAP1 upregulation is associated with the poor prognosis of patients with cancer [205]. The STAT3 signaling pathway accelerates the growth and proliferation of lung cancer cells via miR-33a-5p inhibition and the subsequent activation of karyopherin subunit alpha 4 (KDNA4) [206]. Besides, G-protein alpha-subunit (GNAS) as an upstream mediator can induce the STAT3 signaling pathway through IL-6 to enhance the malignancy and proliferation of cancer cells [207]. So, targeting these two signaling pathways is of importance in cancer therapy. The in vivo and in vitro experiments exhibit that NOB triggers apoptosis and cell cycle arrest. The examination of molecular signaling pathways demonstrates that NOB inhibits the phosphorylation of STAT3, YY1AP1, and SRC/Akt to exert its inhibitory impact on renal carcinoma cells [208]. The tumor microenvironment plays a significant role in the malignant behavior of cancer cells. Hypoxia is a feature of the tumor microenvironment that increases the metastasis of renal carcinoma cells and is associated with recurrence [209,210]. One of the molecular mechanisms involved in metastasis is the EMT [198]. Increasing evidence has shown that hypoxia can trigger the EMT to enhance the migration and invasion of cancer cells [211]. On the other hand, it has been demonstrated that hypoxia, in addition to other well-established inducers, can stimulate oncogenic NF-κB and Wnt/β-catenin signaling pathways [212,213]. These two pathways can function as upstream inducers of the EMT in cancer migration [214,215]. Noteworthy, NOB is capable of suppressing the invasion and migration of renal carcinoma cells. The examination of molecular pathways reveals that NOB downregulates the expression of Wnt and NF-κB to suppress the EMT in the hypoxic condition, leading to decreased the migration and metastasis of cancer cells (Figure 1) [216].

### 5.7. Gastrointestinal Cancers

Colorectal cancer (CRC) is the most common cancer in males and females after lung and breast cancers [217]. A variety of factors contribute to CRC development, such as gender, age, genetic alterations, lifestyle, and inflammatory bowel disease (IBD). The incidence rate of CRC is higher in men compared to women [218,219]. It seems that plant-derived natural compounds are potential agents in CRC chemoprevention [220]. The efficacy of NOB in the treatment of colon cancer is related to its impact on the viability and survival of cancer cells. A metabolite of NOB, known as 4-DMN, and atorvastatin are able to suppress colon cancer malignancy via the stimulation of apoptosis and cell cycle arrest [221]. In this way, NOB enhances the expression of p21, while it reduces the levels of CDK2, CDK4, cyclin D, and cyclin E [222]. It is worth mentioning that inflammatory factors can lead to colon cancer development [218]. During IBD, a number of proinflammatory cytokines such as TNF-α, IL-6, and IL-1β are secreted [223]. These factors are suggested to be involved in colon cancer carcinogenesis, since the downregulation of IL-6 reduced the colon cancer development [223]. The administration of NOB and atorvastatin decreases the levels of proinflammatory cytokines and downregulates the expression of COX-2 to inhibit inflammation-mediated colon cancer development [222]. In fact, in this case, the antitumor activity of NOB results from its anti-inflammatory activity. The epidermal growth factor receptor (EGFR) is a transmembrane glycoprotein that begins several intracellular molecular signaling pathways, leading to cell proliferation and cell growth. β-elemene as an antitumor agent diminishes the migration and invasion of cancer cells by the inhibition of EGFR signaling [224]. The onco-suppressor upstream factors are able to inhibit cancer malignancy via the inhibition of EGFR [225]. These studies exhibit that EGFR signaling is a positive factor for the growth and proliferation of cancer cells, and its targeting is a potential strategy in cancer therapy. A combination of NOB and atorvastatin synergistically suppresses the proliferation and metastasis of colon cancer cells via EGFR downregulation [222]. The Ras homolog gene family member A (RhoA) is a key player of the Ras/Rho superfamily with involvement in different aspects of cells such as proliferation and migration. The abnormal expression of RhoA occurs in different cancers. It is said that the migration and survival of melanoma cells undergo inhibition via RhoA inhibition [226]. The great antitumor activity of lupeol depends on RhoA inhibition to suppress colon cancer capacity in proliferation and growth [227]. In the treatment of colon cancer, NOB negatively affects RhoA expression. The NOB supplementation along with atorvastatin suppresses the invasion and migration of colon cancer cells via RhoA downregulation [222]. In previous sections, we demonstrated that NOB was reported to induce apoptosis in cancer cells through both mitochondrial and ER pathways. The NOB enhances the expressions of ER stress-related proteins such as IRE-1α, ATF4, CHOP, and GRP78. This leads to the stimulation of apoptosis via caspase-4 activation. However, an interesting point is the induction of autophagy by NOB. It seems that the inhibition of autophagy in cancer cells may enhance the number of cells undergoing apoptosis [228]. In gastric cancer cells exposed to NOB, the inhibition of autophagy elevates the capability of NOB in the stimulation of apoptosis [229]. So, in order to enhance the antitumor activity of NOB, autophagy inhibitors such as rapamycin and chloroquine can be used to inhibit protective autophagy and maximize the efficacy of NOB in the elimination of cancer cells.

### 5.8. Hematological Cancers

The c-kit, known as CD117, encoded by the kit gene, is considered as an oncogene factor. The c-kit phosphorylates plasma membrane prohibitin (PHB) at tyrosine259 to ensure the invasion and migration of cancer cells and induces their resistance into chemotherapeutic agents [230,231]. The combination of irinotecan and tankyrase inhibitors diminishes the proliferation and growth of cancer cells via downregulation of the c-kit [232]. The microRNA (miR)-664, as an onco-suppressor, reduces the expression of the c-kit to suppress the proliferation and invasion of cancer cells [233]. In acute myeloid leukemia (AML) cells, NOB targets the c-kit. It appears that the viability and survival of AML cells undergo a decrease by NOB via reducing expression of the c-kit. Notably, a combination of NOB and cytarabine, a chemotherapeutic agent, remarkably decreases c-kit expression in AML therapy [234].

### 5.9. Anti-angiogenesis Effect

**Figure 2:**
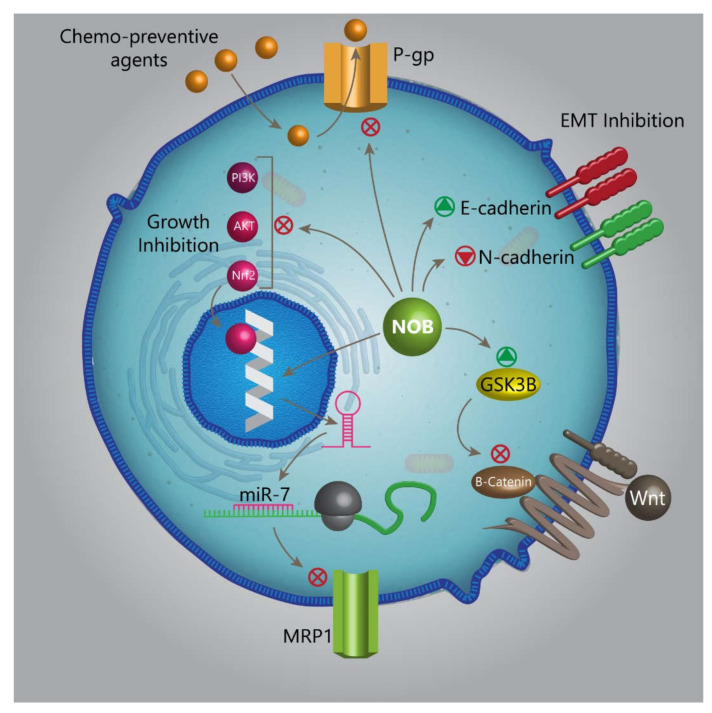
The capability of NOB in targeting various molecular pathways and mechanisms, making it an appropriate option in cancer therapy. P-gp, P-glycoprotein; NOB, nobiletin; miR, microRNA; MRP1, multidrug-resistance-associated protein 1; GSK-3β, glycogen synthase kinase 3 beta; Nrf2, nuclear factor erythroid 2-related factor 2; Akt, protein kinase B; PI3K, phosphatidylinositide-3 kinase; and EMT, epithelial-to-mesenchymal transition.

The process of sprouting new blood vessels from pre-existing ones is defined as angiogenesis [235]. This process is active during embryogenesis, and in adulthood, angiogenesis is transiently activated, for instance, during the reproductive cycle in females. Although angiogenesis seems to be vital for physiological conditions, its activation occurs in a variety of disorders, particularly cancer [236]. Molecularly, vascular endothelial growth factor (VEGF) plays a major role during angiogenesis, and in this way, it interacts with the epidermal growth factor (EGF) and basic fibroblast growth factor (bFGF) [237]. Upstream mediators target these molecular pathways to regulate angiogenesis. The steroid receptor coactivator (Src) and focal adhesion kinase (FAK) are tyrosine kinases capable of controlling angiogenesis. The EGFR effect on migration relies on FAK [238]. Src has been displayed to induce angiogenesis to elevate the growth and migration of cancer cells [239]. Src and EGFR regulate VEGF in angiogenesis by targeting STAT3 [240,241,242,243,244,245]. So, complicated signaling pathways are involved in the regulation of angiogenesis. The administration of NOB inhibits EGFR to downregulate the expression of its downstream targets, including Scr, FAK, and STAT3 (the Src/FAK/STAT3 signaling pathway). As a consequence, the nuclear translocation of STAT3 is inhibited, and its attachment into paxillin is suppressed, leading to the downregulation of angiogenesis and invasion and migration of breast cancer cells (Figure 2, Table 2) [246].

## 6. Conclusion and Remarks

NOB is a naturally occurring compound with potential therapeutic effects that are shown in Table 1. However, we allotted this review to the antitumor activity of NOB. NOB can be used as a chemosensitizer. To date, studies have revealed that NOB can reduce the resistance of cancer cells into chemotherapeutic agents such as cisplatin, PTX, and OX. The chemoprevention impact of NOB is related to its effect on five distinct molecular pathways and mechanisms. The first molecular mechanism is P-gp, which, by inhibition of its activity, NOB paves the road into the penetration of chemo-preventive agents into cancer cells. Second, NOB inhibits the Nrf2/PI3K/Akt pathway to inhibit the growth of cancer cells. Third, NOB reduces the malignant behavior of cancer to sensitize them into chemotherapy via EMT inhibition. Fourth, NOB inhibits the Wnt/β-catenin signaling pathway via GSK-3β upregulation. Fifth, NOB enhances the expression of miR-7 to inhibit MRP1. In the induction of apoptosis, NOB affects various molecular pathways. One of them is the PARP2/SIRT1/AMPK axis. NOB inhibits PARP2 to stimulate the SIRT1/AMPK axis, leading to apoptotic cell death. One of the most important effects of NOB is its capability in suppressing the migration and invasion of cancer cells. In this way, NOB downregulates NF-κB, Wnt, TGF-β, Snail, Slug, and ZEB1 as upstream mediators of EMT, resulting in the reduced metastasis of cancer cells. In addition to EMT, NOB can inhibit MMP-2 and MMP-9 expressions to suppress the metastasis of cancer cells. It is worth mentioning that NOB can target the metabolism of cancer cells, so that it diminishes the aromatase activity, as a factor involved in the growth of breast cancer cells. NOB supplementation induces pro-survival autophagy. It seems that using autophagy inhibitors such as rapamycin and chloroquine enhances the efficacy of NOB in the stimulation of apoptosis. In the induction of apoptosis, NOB targets both the mitochondrion and ER. In inhibition of the migration of cancer cells during hypoxic conditions, NOB downregulates the expressions of NF-κB and Wnt signaling pathways. In respect to the role of inflammation in colon cancer carcinogenesis, NOB reduces the levels of inflammatory factors such as ILs and IFN to suppress cancer development and progression. The interesting point is that NOB exerts an anti-angiogenesis impact by the inhibition of STAT3 and VEGF. Several directions appear to be beneficial about NOB. Based on the minimal side-effects of NOB, it can be applied in clinical trials, and until now, there has been no research in this case. Besides, using different strategies such as nanocarriers seems to be advantageous in enhancing the antitumor activity of NOB via promoting its bioavailability.

## Figures and Tables

**Table 1:** Therapeutic and biological activities of nobiletin (NOB).

Disease/Protective Effect	In Vitro/In Vivo	Dose	Duration of Experiment	Administration Route	Results	References
Cardioprotective	In vivo (rat)	15 mg/kg	Before coronary microembolization	Tail vein	Downregulation of apoptosis and protecting against myocardial injury by induction of PI3K/Akt signaling pathway.	[82]
Cardioprotective	In vitro (human aortic valves)	10, 20, and 50 μM	24 and 48 h	-	By activation of ABCG2 and AKR1B1, NOB suppresses tumor necrosis factor (TNF)-mediated calcification of the human aortic valve.	[83]
Cardioprotective	In vitro (H9c2 cardiomyocytes)	12.5, 25, 50, and 100 μM	24 h	-	Reducing apoptosis and oxidative stress after ischemic/reperfusion (I/R) injury via the stimulation of Akt/GSK-3β.	[84]
Neuroprotective	In vivo (mice)	100 mg/kg/day	6 weeks	Oral gavage	Decreasing the levels of anti-inflammatory cytokines such as TNF-α and interleukin (IL)-1β by downregulation of the NF-κB signaling pathway and, also, inhibition of microglial activation.	[85]
Osteoarthritis	In vitro (primary human chondrocytes) In vivo (mice model of osteoarthritis)	20, 40, and 80 μM 20 mg/kg	2 h 8 weeks	Gavage	Alleviation of osteoarthritis by downregulation of PI3K/Akt/NF-κB pathway and reducing inflammatory factors.	[86]
Antihypertensive	In vivo (rat)	20 and 40 mg/kg	2 weeks	-	Attenuation of vascular changes, induction of antihypertensive effect, inhibition of matrix metalloproteinases (MMP)-2 and -9, and reducing oxidative stress through Nrf2 activation.	[87]
Anti-inflammation	In vitro (human mesangial cells)	5, 10, 20, and 30 μM	24 h	-	By inhibition of STAT3, NOB downregulates the expression of NF-κB to decrease the levels of TNF-α, IL-6, and IL-1β.	[88]
Anti-inflammation	In vitro (macrophages)	0, 10, 20, 40, and 80 μM	24 h	-	NOB enhances the expression of miR-590 to decrease the levels of proinflammatory cytokines.	[89]
I/R injury	In vivo (mice)	5 mg/kg	At the start of reperfusion	Intraperitoneal	Alleviation of hepatic I/R injury by stimulation of autophagy and mitochondrial biogenesis via the SIRT1/FOXO3a axis.	[90]

**Table 2:** The antitumor activity of nobiletin in different cancers.

Cancer Type	Cell Line	In Vitro/In Vivo	Dose	Duration of Experiment	Administration Route	Results	References
Breast cancer	Human breast carcinoma MDA-MB-231 cells	In vitro	0, 10, 30, and 50 μM	24 h	-	Significantly decreasing the expressions of genes related to the malignant behavior of cancer cells such as CXCR4, MMP-9, NF-κB, and MAPK.	[247]
Breast cancer Colon cancer	MDA-MB-435, MCF-7 (human ductal breast carcinoma and adenocarcinoma, respectively), and HT-29 (human colorectal adenocarcinoma) cell lines	In vitro	0, 50, 100, 150, and 200 μM	12, 24, 48, 72, and 96 h	-	Induction of the G1 cell cycle arrest not apoptosis in cancer cells.	[248]
Breast cancer	MCF7 cells	In vitro	0, 1, 5, and 10 μM	0, 3, 6, 9, and 24 h	-	The CYP1A1 induces the bioactivation of NOB in breast cancer cells, resulting in cell cycle arrest at the G1 phase.	[154]
Breast cancer	Three subtypes of breast cancer cell lines, including hormone receptor (ER/PR)-positive MCF-7, hormone receptor-negative but HER2-positive SK-BR-3, and triple-negative MDA-MB-468	In vitro	100 μM	0, 2, 6, 12, and 24 h	-	The stimulation of apoptosis and cell cycle arrest via the downregulation of Bcl-XL, ERK1/2, cyclin D1, Akt, and mTOR and upregulation of p21 and Bax.	[249]
Hepatocellular carcinoma	SMMC-7721 cells	In vitro In vivo	2-128 mg/L 125, 250, and 500 mg/kg	48 h 11 days	Intragastric gavage	Stimulation of the G2 cell cycle arrest, downregulation of Bcl-2 and COX-2, upregulation of Bax and caspase-3, and triggering apoptosis.	[250]
Liver cancer	HepG2 cells	In vitro	0.5, 1, and 2.5 μM	12 h	-	Suppressing the invasion and migration of cancer cells via the downregulation of ERK and the PI3K/Akt signaling pathway.	[251]
Hepatocellular carcinoma Neuroblastoma cells	HuH-7 human hepatocarcinoma cells and SK-N-SH human neuroblastoma cells	In vitro	100 μM	24 h	-	Increasing the levels of genes related to the endoplasmic reticulum, such as CHOP, Ddit3, Trib3, and Asns, and decreasing the levels of genes related to cell cyclins, such as Ccna2, Ccne2, and E2f8.	[252]
Gastric cancer	Four human gastric cancer cell lines, including TMK-1, MKN-74, KATO-III, and MKN-45	In vitro	0, 50, 100, 150, 200, and 250 μM	24 h	-	Induction of apoptosis and the cell cycle arrest and enhancing the chemotherapy efficacy of cisplatin.	[253]
Gastric cancer	AGS, MKN-45, SNU-1, and SNU-16 cells	In vitro	0, 12.5, 25, 50, 100, and 200 μM	48 h	-	Stimulation of the G1 cell cycle arrest and apoptosis via enhancing the levels of the Bax/Bcl-2 ratio, caspase-3, caspase-9, and PARP.	[254]
Gastric carcinoma	Human AGS gastric adenocarcinoma cell line	In vitro	0, 1, 1.5, and 2 μM	24 and 48 h	-	Stimulation of a diminution in the invasion and migration of gastric cancer cells via the inhibition of a small GTPase signal and FAK/PI3K/Akt.	[255]
Glioma	Human U87 and Hs683 glioma cell lines	In vitro	20, 50, and 100 μM	24 and 48 h	-	Suppressing the migration, invasion, and proliferation of cancer cells by induction of the cell cycle arrest (downregulation of cyclin D1 and cyclin-dependent kinase-2) and inhibition of the MAPK and Akt signaling pathways.	[256]
Lung cancer	A549 and H460 cell lines	In vitro In vivo	20, 40, and 80 μM 600 μg	24 h 30 days	Intraperitoneal	Induction of the G1 cell cycle arrest and subsequent sensitivity of cancer cells to paclitaxel and carboplatin.	[257]
Nasopharyngeal carcinoma	HONE-1 and NPC-BM, human NPC cells lines	In vitro	0, 10, 20, and 40 μM	12 and 24 h	-	Reducing the expression of MMP-2 and suppressing the phosphorylation of ERK1/2 mediate the antitumor activity of NOB against cancer cells.	[258]
Ovarian cancer	Human ovarian cancer cell lines, OVCAR-3 and A2780/CP70	In vitro	0, 5, 10, 20, 40, 80, and 160 μM	16 h	-	Simultaneously reducing the levels of HIF-1α, Akt, and NF-κB, leading to the downregulation of VEGF and the subsequent inhibition of angiogenesis.	[259]
Prostate cancer	PC-3 cells	In vitro	0, 5, 10, 20, 40, 80, and 160 μM	24 h	-	The downregulation of Akt by NOB impairs the proliferation and growth of cancer cells. By the inhibition of Akt, the expression of HIF-1α as a downstream target undergoes a decrease.	[260]
Fibrosarcoma	Human fibrosarcoma HT-1080 cells	In vitro	16, 34, and 64 μM	24 h	-	Inhibiting the metastasis and migration of cancer cells through the downregulation of MEK.	[261]
Fibrosarcoma	Human fibrosarcoma HT-1080 cells	In vitro	64 μmol/L	12 h	-	Reducing the expression of pro-MMPs and enhancing the expression of the tissue inhibitor of metalloproteinase 1. Suppressing the activity of MEK1/2 and inducting the phosphorylation of JNK.	[262]

## Abbreviations

NOB, nobiletin; PMF, polymethoxyflavone; DMN, demethylnobiletin; CAGE, choline and geranic acid; DM, diabetes mellitus; TNF, tumor necrosis factor; ROS, reactive oxygen species; RA, rheumatoid arthritis; MMPs, matrix metalloproteinases; Nrf2, nuclear factor erythroid 2-related factor 2; SOD, superoxide dismutase; HO-1, heme ocygenase-1; NQO1, NADPH quinone reductase; I/R, ischemic/reperfusion; CAT, catalase; GSH-PX, glutathione-peroxidase; MAPK, mitogen activated-protein kinase; MDR, multidrug resistance; P-gp, P-glycoprotein; ABC, ATP-binding cassette; PTX, paclitaxel; RTK, receptor tyrosine kinase; FDA, Food and Drug Administration; MRP1, multidrug resistance-associated protein 1; MYCN, neuroblastoma-derived MYC; FSIP1, fibrous sheath-interacting protein 1; OX, oxaliplatin; PARP, poly (ADP-ribose) polymerase; SIRT1, sirtuin 1; EMT, epithelial-to-mesenchymal transition; E1, estrone; TEB, tumor-educated B; LAMA4, laminin subunit alpha 4; PCD, programmed cell death; TLRs, Toll-like receptors; NK, natural killer; PAMPs, pathogen-associated molecular patterns; LPS, lipopolysaccharide; IFN, interferon; ER, endoplasmic reticulum; UPR, unfolded protein response; ERAD, ER-associated degradation; PERK, PKR-like ER-associated kinase; IRE1α, inositol requiring enzyme-1α; ATF6, activating transcription factor 6; STAT3, signal transducer and activator of transcription 3; YY1AP1, YY1-associated protein 1; KDNA4, karyopherin subunit alpha 4; GNAS, G-protein alpha-subunit; CRC, colorectal cancer; IBD, inflammatory bowel disease; EGFR, epidermal growth factor receptor; RhoA, Ras homolog family, member A; PHB, prohibitin; miR, microRNA; AML, acute myeloid leukemia; ECM, extracellular matrix; SP-1, specificity protein-1; CREB, cAMP response element-binding protein; VEGF, vascular endothelial growth factor; EGF, epidermal growth factor; bFGF, basic fibroblast growth factor; Src, steroid receptor coactivator; FAK, focal adhesion kinase; and PXN, paxillin.
